# Insecure adult attachment styles are associated with parental reflective functioning pre-mentalizing modes

**DOI:** 10.1192/j.eurpsy.2023.477

**Published:** 2023-07-19

**Authors:** L. Erkoreka, G. Urrutia

**Affiliations:** ^1^Galdakao-Usansolo Hospital, Galdakao; ^2^University of the Basque Country UPV/EHU, Leioa; ^3^Biocruces Bizkaia HRI, Barakaldo, Spain

## Abstract

**Introduction:**

Parental Reflective Functioning (PRF) refers to parents’ capacity to view their child’s and their own behavior considering inner mental states, like thoughts, desires, and intentions. It has been related to attachment, mentalizing capacities, and psychopathology in children. An association between adult attachment style and reflective functioning has been described. Studies have also suggested that parental insecure attachment is related to lower levels of PRF.

**Objectives:**

We aim to study the association between specific adult attachment styles (anxious [Anx] and/or avoidant [Av]) and specific PRF modes (pre-mentalizing [PM], certainty about mental states [CMS] and/or interest and curiosity [IC]).

**Methods:**

A sample of 238 parents (228 mothers and 34 fathers) of 263 children aged 0 to 5 years old were recruited through informal difussion of the study in social media. These parents completed the Experiences in Close Relationships-Revised (ECR-R) and the Parental Reflective Functioning Questionnaire-18 (PRFQ-18) online. Sex and age of parents and children were also gathered. In the first place, Pearson’s correlation was conducted to study the association between the ECR-R and the PRFQ-18 subscales. In a second step, general linear models were used to control for the effect of sex and/or age, when necessary.

**Results:**

Mothers’ mean age was 35,59±4,55 and fathers’ 38,26±4,47. Among children, a total of 119 were girls (45,2%) and 140 (53,2%) boys; in 4 (1,5%) cases the sex was not specified. The association between attachment and PRF subscales is shown in Table 1.

**Table:** 

	1	2	3	4	5	6	7	8
Parent age	-							
Parent sex	**-0,196** **	-						
Child age	**0,273** **	0,054	-					
Child sex	0,062	0,014	0,041	-				
PRFQ PM	0,120	-0,059	0,072	-0,001	-			
PRFQ CMS	-0,109	**0,135** *	0,137*	0,015	**-0,256** **	-		
PRFQ IC	-0,099	**0,163** **	0,121	-0,069	-0,072	0,078	-	
ECR-R Anx	0,115	**-0,138** *	-0,027	0,006	**0,410** **	**-0,232** **	-0,035	-
ECR-R Av	0,137*	-0,049	0,007	-0,091	**0,186** **	-0,049	-0,103	**0,279** **

** p<.001,

* p<.05

In a second step, the influence of Anx attachment on CMS was controlled for parents’ sex and children’s age; β-value of Anx was -0,288 (p<.000) and the whole model explained 70% of the variability of CMS.

**Conclusions:**

We observed that Anx attachment is associated with lower CMS and greater PM. With regard to CMS, both high and low extreme scores have been proposed to be less adaptative than average scores. Av attachment has also been related to higher PM scores. PM mode, which involves “an inability to hold the child’s mental states in mind and/or to have malevolent attributions about the child’s behavior”, is indicative of pathological PRF, and seems to be associated with insecure attachment (↑Anx/Av). Our results are in line with previous works (San Cristobal et al. Front. Psychol. 2017; Luyten et al. PLOS ONE 2017), and suggest that PRF could play a role in the intergenerational transmission of attachment, which should be further investigated.

**Disclosure of Interest:**

None Declared

